# Structural Attributes and Principles of the Neocortical Connectome in the Marmoset Monkey

**DOI:** 10.1093/cercor/bhab191

**Published:** 2021-07-19

**Authors:** Panagiota Theodoni, Piotr Majka, David H Reser, Daniel K Wójcik, Marcello G P Rosa, Xiao-Jing Wang

**Affiliations:** Center for Neural Science, New York University, New York, NY 10003, USA; New York University Shanghai, Shanghai 200122, China; NYU-ECNU Institute of Brain and Cognitive Science at New York University Shanghai, Shanghai 200062, China; Laboratory of Neuroinformatics, Nencki Institute of Experimental Biology of Polish Academy of Sciences, Warsaw 02-093, Poland; Australian Research Council, Centre of Excellence for Integrative Brain Function, Monash University Node, Clayton, VIC 3800, Australia; Neuroscience Program, Biomedicine Discovery Institute and Department of Physiology, Monash University, Clayton, VIC 3800, Australia; Australian Research Council, Centre of Excellence for Integrative Brain Function, Monash University Node, Clayton, VIC 3800, Australia; Graduate Entry Medicine Program, Monash Rural Health-Churchill, Monash University, Churchill, VIC 3842, Australia; Laboratory of Neuroinformatics, Nencki Institute of Experimental Biology of Polish Academy of Sciences, Warsaw 02-093, Poland; Australian Research Council, Centre of Excellence for Integrative Brain Function, Monash University Node, Clayton, VIC 3800, Australia; Neuroscience Program, Biomedicine Discovery Institute and Department of Physiology, Monash University, Clayton, VIC 3800, Australia; Center for Neural Science, New York University, New York, NY 10003, USA

**Keywords:** allometric scaling, distance-dependent structural connectivity, hierarchy, network, primate

## Abstract

The marmoset monkey has become an important primate model in Neuroscience. Here, we characterize salient statistical properties of interareal connections of the marmoset cerebral cortex, using data from retrograde tracer injections. We found that the connectivity weights are highly heterogeneous, spanning 5 orders of magnitude, and are log-normally distributed. The cortico-cortical network is dense, heterogeneous and has high specificity. The reciprocal connections are the most prominent and the probability of connection between 2 areas decays with their functional dissimilarity. The laminar dependence of connections defines a hierarchical network correlated with microstructural properties of each area. The marmoset connectome reveals parallel streams associated with different sensory systems. Finally, the connectome is spatially embedded with a characteristic length that obeys a power law as a function of brain volume across rodent and primate species. These findings provide a connectomic basis for investigations of multiple interacting areas in a complex large-scale cortical system underlying cognitive processes.

## Introduction

Cognitive processes involve multiple interacting brain areas. However, the underlying architecture for interareal interactions, represented by neuronal connections, is not yet fully understood. Given current progress toward large-scale, simultaneous recordings from many areas, there is an even greater need to understand the principles of neural connectivity, in order to enable mechanistic interpretation of the emerging patterns of activity.

The last decade has seen a rapid change in neuroanatomy, from descriptive studies focused on few areas and nuclei at a time to those aimed at identifying the organizational principles, based on comprehensive and quantified large-scale datasets. Although studies in mice have been at the forefront of this effort (e.g., Gămănuţ et al. 2018), translation to principles applicable to the human brain also requires knowledge of the network properties of the nervous system in other mammals, including in particular nonhuman primates (e.g., Van Essen and Glasser 2018). For example, the primate prefrontal cortex has expanded and become more complex through the addition of new areas (Mansouri et al. 2017), and some of the networks of brain areas that are involved in high-order cognitive processes (and are affected in psychiatric conditions) differ significantly from those found in rodents (Schaeffer et al. 2020). Moreover, primates have a large portion of the cortex devoted to vision, including many areas devoted to fine recognition of objects and to the complex spatial analyses required for oculomotor coordination (Solomon and Rosa 2014). The auditory cortex is similarly specialized, including a network of areas for identifying and localizing vocalizations (Miller et al. 2016), whereas the motor cortex contains a unique mosaic of premotor areas for planning and executing movements (Bakola et al. 2015). Therefore, to facilitate translation of discoveries in animal models to improvements in human health, studies of nonhuman primates are crucial to fill the gap between rodent and human models.

Macaques are the nonhuman primate genus for which the most comprehensive knowledge of the connectional network of the cortex has been achieved, initially by studies based on meta-analyses of the literature (Bakker et al. 2012), and more recently by retrograde tracer injections obtained with a consistent methodology (Markov et al. 2013, 2014a, 2014b). Analyses of macaque and mouse data have already highlighted putative organizational principles of the mammal cortical mesoscale connectome (Ercsey-Ravasz et al. 2013; Song et al. 2014; Horvát et al. 2016; Gămănuţ et al. 2018). However, extrapolating from any single species to human is problematic without knowledge of the scaling rules that govern anatomical similarities and differences (Chaplin et al. 2013).

The marmoset is a nonhuman primate model with characteristics that complement those of the macaque in terms of facilitating analyses of brain anatomy, development, and function. Marmosets have a relatively short maturation cycle, which facilitates the development of transgenic lines and studies across the life span (Sasaki 2015). At the same time, the key anatomical features that motivate studies of the macaque brain are present (Kaas 2021), including networks of frontal, posterior parietal, and temporal association cortex (Palmer and Rosa 2006a; Reser et al. 2009, 2013; Burman et al. 2011, 2014). The volume of the marmoset brain is approximately 12 times smaller than that of the macaque brain, which in turn is 15 times smaller than the human brain, offering potential insights on scaling properties of the cortical network.

Here, we provide the first account of the statistical properties of the marmoset cortical connectome, taking advantage of an online database of the results of retrograde tracer injections into 55 (out of the 116) cortical areas currently recognized for this species (Majka et al. 2020). The dataset consists of connectivity weights, laminar origin of the projections and wiring distances. This allowed us to explore the statistical properties of the cortico-cortical connections and the architecture of the connectome by defining its hierarchical organization, and the characteristics of its spatial embedding. Furthermore, we studied how microstructural properties within each cortical area relate to the hierarchical organization of the connectome, providing a direct link to different scales within the cortex. In addition, we note conserved properties of the cortico-cortical connections across species, as well as differences that are species, or brain size, dependent. Finally, we present an allometric scaling law of the spatial localization of the connections with brain size, which enables us to extrapolate this connectional attribute to humans.

## Materials and Methods

### Connectivity Data

**Figure 1:**
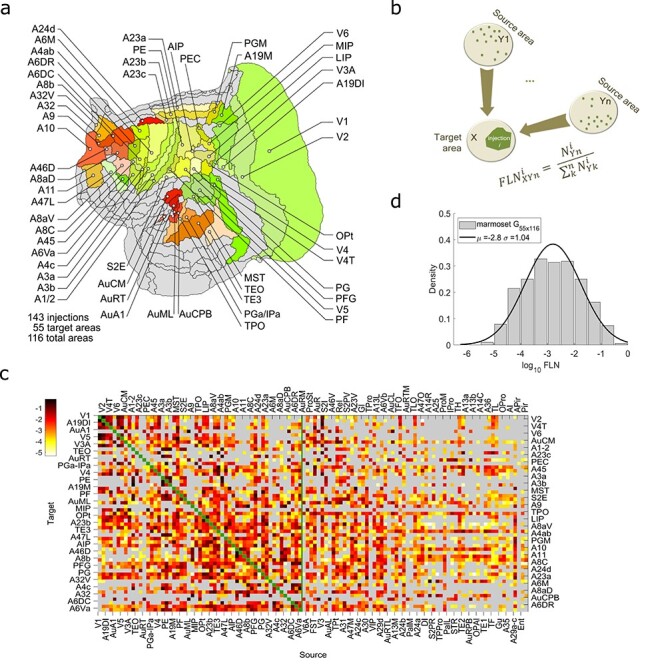
Cortico-cortical connectivity weights. (*a*) The analysis is focused on 55 cortical areas highlighted in different colors on the 2-dimensional flattened map of the marmoset cortex. The gray shaded areas are those for which no tracer injection was available. (*b*) Schematic description of the FLN found in area $Yn$ after the retrograde tracer injection $i$ in the cortical area $X$ (${\mathrm{FLN}}_{XYn}^i$). (*c*) The weighted and directed marmoset cortical interareal connectivity matrix. The rows are the 55 target areas and the columns the 116 source areas that provide inputs to the target areas. Each entry in the matrix is the base 10 logarithm of the arithmetic mean of the FLN $({\log}_{10}\mathrm{FLN})$ across injections within the same target area. Gray: absence of connections, green, along the diagonal line: presence of intra-area connections (they have not been quantitively measured and the corresponding FLN is set to 0). The vertical green line defines the limit of the edge complete $55\times 55$ subnetwork in which all inputs and outputs are known. (*d*) The distribution of the connectivity weights, shown in (*c*), reveals that the connectivity weights are highly heterogenous, they span 5 orders of magnitude, and they are log-normally distributed. Bin size = 0.5 on logarithmic scale. The black line is Gaussian fit to the ${\log}_{10}\mathrm{FLN}$ values.

The marmoset connectivity data consist of the first large-scale cortico-cortical connectivity dataset, which is available through the Marmoset Brain Connectivity Atlas portal (http://marmosetbrain.org). The detailed methods regarding data collection have been described elsewhere (Majka et al. 2016, 2020). In brief, 143 retrograde tract-tracing experiments were performed in 52 young adult (1.3–4.7 years) common marmosets (*Callithrix jacchus*; 31 male and 21 female), using 6 types of retrograde tracers: DY (diamidino yellow, 35 injections), FR (fluororuby: dextran-conjugated tetramethylrhodamine, 35 injections), FB (Fast blue, 29 injections), FE (fluoroemerald: dextran-conjugated fluorescein, 23 injections), and CTBgr and CTBr (cholera toxin subunit B, conjugated with Alexa 488 or Alexa 594 (12 and 9 injections, respectively)). The centers of these injections were located in 55 cortical areas (Fig. 1*a*), some of which received more than one injection (Supplementary Tables 1, 2, and3). All experiments conformed to the Australian Code of Practice for the Care and Use of Animals for Scientific Purposes and were approved by the Monash University Animal Experimentation Ethics Committee (Majka et al. 2020). The use of retrograde tracers allowed quantized visualization of individual cell bodies and their precise location relative to cortical layers, which subsequently allowed for precise quantification of the labeled cells. Each injection of a retrograde tracer in a cortical area (named as *target area*) results in labeling the neurons that project to it. It has been shown that the majority of the projections to the injected site stem from within the same cortical area (Markov et al. 2011). Similarly, in the marmoset, most of the projections are from within the injected area, but these intrinsic connections are not considered here, as the focus is primarily on interareal networks. Based on the parcellation under consideration (Paxinos et al. 2012) the labeled neurons found in each cortical area (referred to as *source areas*) were counted and categorized based on their laminar position. If the labeled neurons were located above the center of layer 4, they were categorized as supragranular neurons, and infragranular neurons otherwise. At the same time, the stereotaxic coordinates of each cell were recorded to allow area-independent analyses (Majka et al. 2020).

By normalizing the number of labeled neurons in each cortical area (other than the target area) with the total number of labeled neurons in all cortical areas (except the target area) in the same hemisphere, we obtained the “fraction of extrinsic labeled neurons (FLNe, or FLN for simplicity),” which represents the connection weight from the source area to the target area (Markov et al. 2014a). Specifically, if $X$ is an injected cortical area with a retrograde fluorescent tracer, then the fraction of labeled neurons (FLN) found extrinsic to it, for example in area $Y$, is defined as $\mathrm{FLN}e(X \leftarrow Y)\equiv{\mathrm{FLN}}_{XY}=\frac{\mathrm{number}\ \mathrm{of}\ \mathrm{labeled}\ \mathrm{neurons}\ \mathrm{in}\ \mathrm{area}\ Y}{\mathrm{total}\ \mathrm{number}\ \mathrm{of}\ \mathrm{extrinsic}\ \mathrm{labeled}\ \mathrm{neurons}}$, (Fig. 1*b*). The ${\mathrm{FLN}}_{XY}$ can be interpreted as the probability of an extrinsic labeled neuron that projects into the target area $X$, being in area $Y$. In Figure 1*b*, the arithmetic average value of the FLN for each target-source pair across injections within the same target area is shown. The bars in the density plot in Figure 1*d* (as well as in all density plots accordingly) are the counts of ${\log}_{10}\mathrm{FLN}$ values falling in each bin, divided by the bin size (bin size = 0.5) and by the total number of the nonzero FLN values (3474 out of 55 × 116 = 6380 in total possible interareal connections were present). Within the injected area the FLN value is set to zero, and therefore also excluded from the density plot. The line is the maximum likelihood Gaussian fit on the ${\log}_{10}\mathrm{FLN}$ values.

### Network-Related Properties

**Figure 2:**
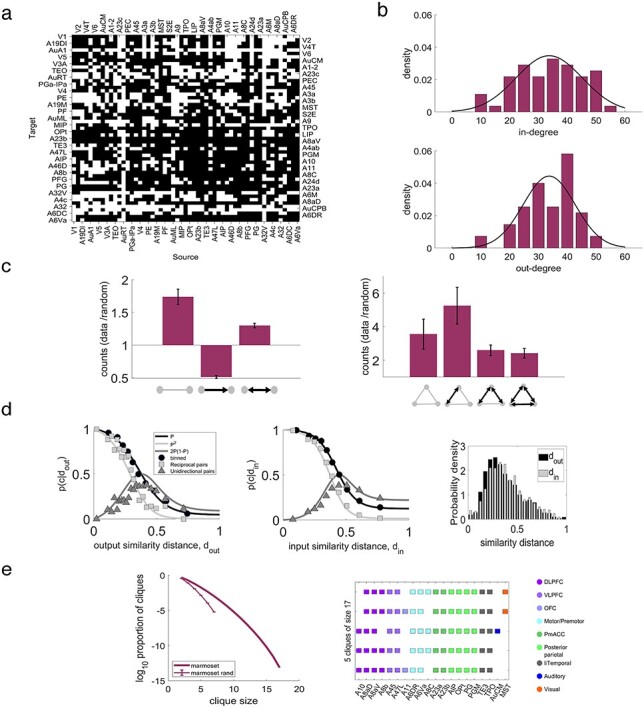
Marmoset network connectivity properties. (*a*) The edge-complete subnetwork, in which all inputs and outputs are known, shows a dense matrix of topological connections. Black: existence, white: absence of a connection. (*b*) In- (top) and out- (bottom) degree distribution of the target areas. Gray lines are Gaussian fits to the data. (*c*) Average fraction of 2- (left) and 3- (right) node motif counts of the edge-complete subnetwork to the 2- and 3-node motif counts, respectively, of a randomized version of the edge-complete network keeping the in- and out-degree the same across 100 realizations. Error bars are one standard deviation of these fractions. (*d*) Proportion of connection as a function of the output (left) and input (middle) similarity distance. Black circles are the number of present connections divided by the number of possible connections in the distance bin. Black line is maximum likelihood fit on the unbinned data. Light gray line is prediction for the reciprocal pairs from the fitted black plot and light gray squares are the proportions of reciprocal pairs in the given bin. Dark gray line is the prediction for the unidirectional pairs and dark gray triangles are the proportions of unidirectional pairs in the given bin. Right: Distribution of the output (black) and input (gray) similarity distances. (*e*) Left: Base 10 logarithm of the proportion of cliques as function of the clique size in the data and the average proportion of cliques in 1000 realizations of a random network of same size where the in- and out- degree sequences are the same as in the data (error bars are one standard deviation). Right: 5 cliques of size 17, combinedly formed by 20 areas that constitute the core of the marmoset cortical connectome.

For the topological properties of the connectome, we binarized the FLN connectivity matrix (Fig. 1*c*) by assigning the value 1 (presence of connection) when $\mathrm{FLN}>0$ and 0 (absence of connection) otherwise, and considered the edge-complete $N\times N$  $(N=55)$ network where all inputs and outputs are known (Fig. 2*a*). The “in-degree” of an area *X* (${k}_X^{\mathrm{in}}$) is the number of inputs to this area, meaning the number of areas that project to it. The “out-degree” of an area *X* (${k}_X^{\mathrm{out}}$) is the number of outputs from this area, meaning the number of areas that this area projects to. In Figure 2*b* the density of in- and out- degree of the edge-complete subnetwork (excluding self-connections) was binned, with bin size 5. The height of each bar denotes the counts divided by the bin size and the total number of areas (*N*). The black lines are the maximum likelihood Gaussian fits on the normalized in- and out- degree values. The clique size $k$ is a $k\times k$ subnetwork that is fully connected (100% density). In Figure 2*e*, left, and Supplementary Figure 1*b*, for a clique of size $ \kappa$ we plot the base 10 logarithm of cliques found in the edge-complete network, divided by the maximum number of cliques of size $k$ that could be found in the edge-complete network, by taking the $n\ \mathrm{choose}\ k$ combinations. We plot the same also for the average random network of same size with same in- and out- degree sequences. The probability of connections as a function of similarity distance (Fig. 2*d*) was computed following the same method as in Song et al. (2014)).

### Hierarchical Structure

**Figure 3:**
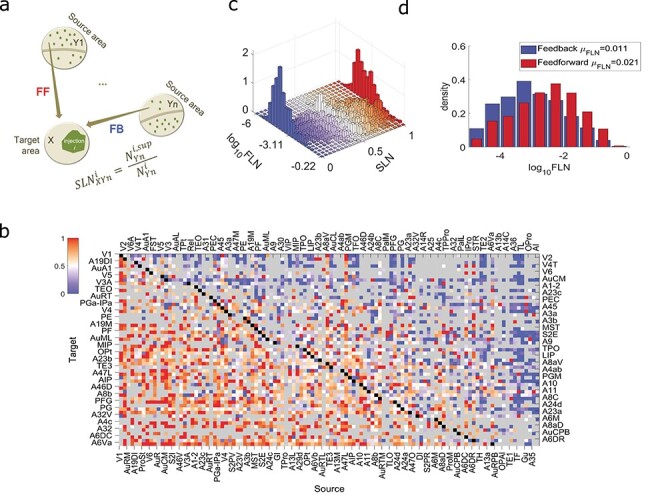
Structural hierarchy. (*a*) Schematic description of the computation of the supragranular labeled neurons found in area $Yn$ after the retrograde tracer injection $i$ in the cortical area $X$ (${\mathrm{SLN}}_{XYn}^i$). Projections with $\mathrm{SLN}>0.5$ (red entries in (*b*)) are considered as FF projections and those with $\mathrm{SLN}<0.5$ are FB projections (blue entries in (*b*)). (*b*) The SLN matrix. The rows are the 55 target areas and the columns the 112 source areas that provide inputs to each target area, ordered according to the computed hierarchy (Fig. 4*a*, Supplementary Fig. 2*b*, right). Each entry in the matrix is the weighted mean supragranular labeled neurons across injections within the same target area. Gray: absence of connections, black: presence of recurrent connections. (*c*) Two-dimensional distribution of the FLN and SLN values. The distribution of SLN is not dependent on the strength of connections, except, as expected, at the edges of the distribution formed by very few labeled neurons. (*d*) Distribution of the FLN values of the FF connections (red; $\mathrm{SLN}>0.5$) and of the FB connections (blue; $\mathrm{SLN}<0.5$, with the first being stronger than the latter (higher mean; the 2 distributions are different (2-sided 2-sample Kolmogorov–Smirnov test: $p=2.58\times{10}^{-40}$, Hedges’ g effect size: $g=0.52$), with different mean (2-sided 2-sample *t*-test: $p=1.93\times{10}^{-43}$) but same variance (2-sided 2-sample *F*-test: $p=0.86$).

The FLN found in the supragranular layers of the source area can be used to calculate the hierarchical rank of each area, and it is related to hierarchical distance (Barone et al. 2000; Markov et al. 2014b; Chaudhuri et al. 2015). This fraction of supragranular labeled neurons (SLN) is given by $\mathrm{SLN}(X \leftarrow Y)\equiv{\mathrm{SLN}}_{XY}=\frac{\mathrm{number}\ \mathrm{of}\ \mathrm{supragranular}\ \mathrm{labeled}\ \mathrm{neurons}\ \mathrm{in}\ \mathrm{area}\ Y}{\mathrm{number}\ \mathrm{of}\ \mathrm{labeled}\ \mathrm{neurons}\ \mathrm{in}\ \mathrm{area}\ Y}$ (Fig. 3*a*), where $X$ is the area injected with retrograde tracer (target area) and $Y$ is the source area whose neurons project to area $X$. In Figure 3*b* the weighted average across injections in the same target area is shown. The areas were ordered with increasing hierarchical index values (Supplementary Fig. 2*b*, right). Areas APir, Pir, Ent, and A29a-c are not shown in the matrix because a layer 4 could not be identified, and therefore the SLN is not defined. In Figure 3*c*, the bars are counts of FLN and SLN within the corresponding bin size (bin size of SLN = 0.05, bin size of ${\log}_{10}\mathrm{FLN}$ = 0.289) divided by the bin sizes of SLN and FLN and the total number of the nonzero SLN values. In Figure 3*d* we categorized the FLN values based on whether they are greater than 0.5, corresponding to a feedforward (FF) projection, or smaller than 0.5, corresponding to a feedback (FB) projection, and plotted the probability density as in Figure 1*d*.

**Figure 4:**
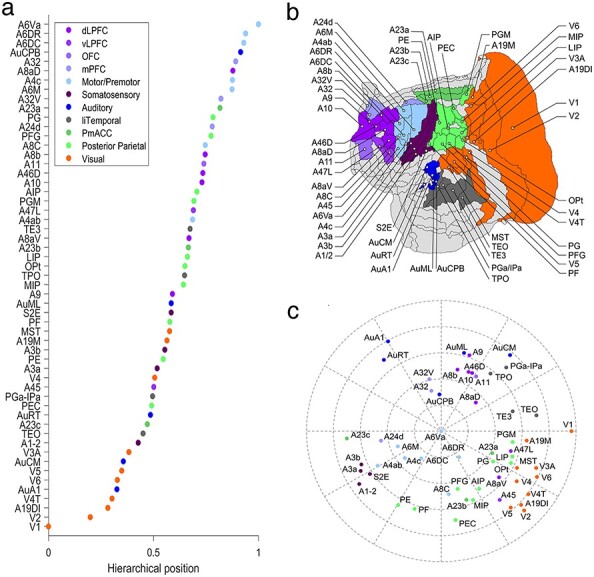
Hierarchical structure. (*a*) Hierarchy of the edge-complete network. (*b*) Flat map of the marmoset cortex, where the color of the shaded areas follows the same coloring scheme as in (*a*). (*c*) Two-dimensional representation of the connectivity strength between areas. The radial direction (distance from the outer edge) is defined by the hierarchical position, and the angular distance is given by the inverse of the strength of the connection. It reveals that functionally related areas are grouped together, sensory areas form parallel streams of processing, and different association areas are related to different sensory modalities.

The hierarchical index for each cortical area *h_i_* (Fig. 4*a*) was computed via a beta-regression model (Cribari-Neto and Zeileis 2010), where for any target-source pair of areas the difference of their indices can predict the SLN in the source area, as was done for the macaque cortical areas (Markov et al. 2014b; Chaudhuri et al. 2015). This relationship is expressed through the following equation: $\mathrm{SLN}(X \leftarrow Y)\approx{g}^{-1}({h}_X-{h}_Y)$, where ${g}^{-1}$ is the logit link function. To obtain the hierarchical indices, we used the model fitting function “betareg” in R software, which results in high correlation between predicted and observed SLN values (Supplementary Fig. 3*c*). Nevertheless, a linear regression model, as in the case of the macaque hierarchy, gives similar results (Supplementary Fig. 3*a*,*b*). In the model, we considered the SLN values of all existing projections from all the injections.

The circular embedding in Figure 4*c* is a polar plot of the target areas ${A}_i$, with radial coordinate $R({A}_i)=\sqrt{1-{h}_i}$ and angular coordinate $\theta ({A}_i)={\theta}_i$, where ${\theta}_i$ is the angle assigned to each area such that $-{\log}_{10}(\mathrm{FLN}({A}_i,{A}_j))=r\ \min (|{\theta}_i-{\theta}_j|,2\pi -|{\theta}_i-{\theta}_j|)$, where *r* is a free parameter, and computed following the same method applied to the macaque cortical areas (Chaudhuri et al. 2015). The angle of area V1 was assigned to be zero, and the system of coordinates was shifted such that the highest area in the hierarchy is at the center of the plot.

### Wiring Distances and Exponential Distance Rule

If *X* and *Y* are 2 cortical areas, then the wiring distance $d\equiv{d}_{X \leftrightarrow Y}$ between them is defined as the shortest path through the white matter, avoiding the gray matter, between their barycenters. The definition is the same as in the studies where the wiring distance of the macaque and mouse was measured and used for the exponential distance rule (EDR) that shows the exponential distribution of the projection length (Ercsey-Ravasz et al. 2013; Horvát et al. 2016). The details of the way the wiring distance were computed can be found in Majka et al. (2020). In brief, the shortest path between the barycenters of 2 areas was computed by simulating 3-dimensional trajectories between the areas, where each voxel in the 3-dimensional template of the marmoset cortex was assigned different viscosity parameters. The fastest trajectory corresponded to the shortest path. The interareal wiring distances were used in Figures 6*a*,*b* and Supplementary Figure 4*b*. For calculations of the EDR (Fig. 6*c* and Supplementary Fig. 5), we used the projection lengths of each labeled neuron, from the injection site to its coordinates measured with the method described above, after projecting them to the mid-thickness surface in order to avoid bias between distances of supragranular and infragranular neurons. In the EDR plots, each bar represents the counts of the projection lengths lying on the bin divided by the total number of projection lengths (1 966 028 in total) including the projections lengths of the labeled neurons found within the injected area. The red plot in Figure 6*b*,*c* is the linear fit to the log bar plot of the projection lengths, as applied in previous studies (Ercsey-Ravasz et al. 2013; Horvát et al. 2016). Similar fits are also drawn in the common template case (Supplementary Fig. 5).

### Local Microstructural Properties

**Figure 5:**
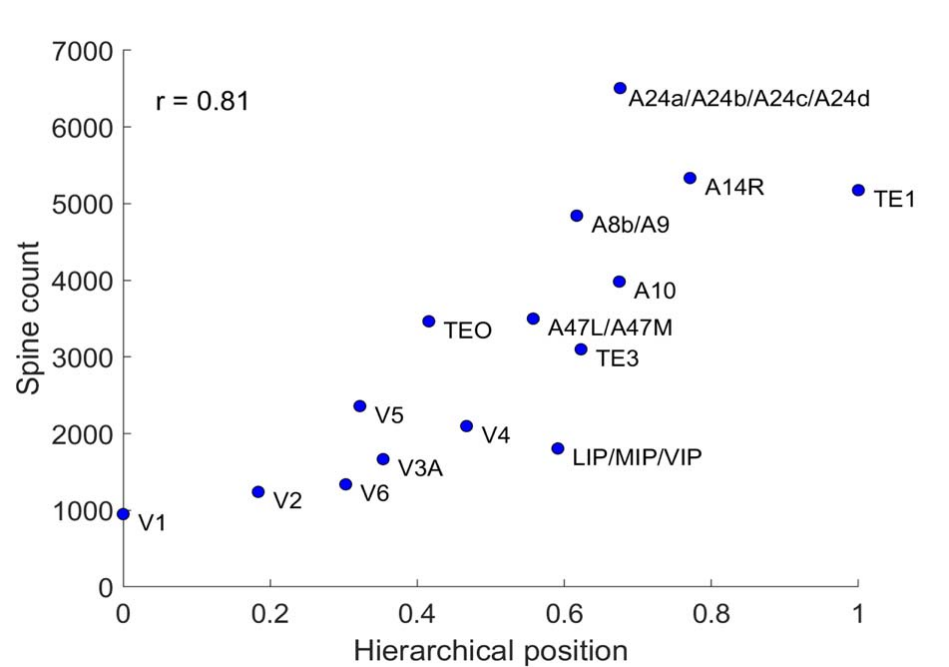
Microstructural properties along the hierarchy. Spine count of basal dendrite in a layer 3 pyramidal neuron is correlated with hierarchical position. *r* is the Pearson correlation.

We extracted the spine counts of neurons in different marmoset cortical areas from studies where a uniform method was used (intracellular injection of Lucifer yellow in fixed slices), and the same types of spines have been measured (those at the basal dendrites of pyramidal neurons in layer III), in marmosets of the same age as the ones of the current study (from 18 months to 4.5 years old). We have collected the spine count for 15 cortical areas based on the nomenclature of the papers, which correspond to 22 cortical areas according to the current (Paxinos et al. 2012) parcellation. Details of the spine count and the corresponding references are shown in Supplementary Table 4 and Supplementary Figure 6. In Figure 5, the hierarchical values of the 22 areas have been normalized to 1 and then averaged among areas that correspond to the same spine count (e.g., the hierarchical index of the area A8b/A9 is the average normalized hierarchical index of areas A8b and A9). In Supplementary Figure 6*b*, we show that if we instead keep the hierarchical rank of each area and duplicate the spine count for the merged areas (e.g., area A8b has the same spine count with area A9 but different hierarchical index) the correlation of the spine count is still high. The brain volumes of the marmoset, macaque, mouse, rat, and human have been obtained from the literature (Zhang and Sejnowski 2000) (Supplementary Table 5).

### Data and Code Availability

The cortico-cortical connectivity datasets analyzed in the current study are available under the terms of Creative Commons Attribution-ShareAlike 4.0 License and publicly available through the Marmoset Brain Connectivity Atlas portal (http://marmosetbrain.org). Software was written in the MATLAB (https://www.mathworks.com/products/matlab.html), R (https://www.r-project.org/), and Python (https://www.python.org/) programming languages, based on the algorithms of the corresponding published articles and are available upon reasonable request.

## Results

### Connectivity Weights are Highly Heterogeneous and Log-Normally Distributed

We have analyzed the results of 143 retrograde tracer injections placed in 52 young adult marmosets (1.3–4.7 years; 31 male, 21 female; Supplementary Tables 1 and 2, available through the Marmoset Brain Connectivity Atlas (http://marmosetbrain.org) (Majka et al. 2020). The center of each injection was assigned to 1 of 55 target areas from the Paxinos 116-area parcellation by a process that is detailed in Majka et al. (2020). The 55 target areas were distributed across the marmoset cortex (Fig. 1*a* and Supplementary Table 6). The use of retrograde tracers allows quantification of the number of neurons that project from 115 potential source areas to a given target area.

A quantitative measure of the connectivity weight from each source area to a target area (Fig. 1*b*) is defined as the number of projection neurons found in each source area divided by the total number found across all source areas in the same hemisphere, called the FLN. This analysis, which excluded connections from cells located in the same cytoarchitectural area (intrinsic connections), resulted in a $55\times 116$ connectivity matrix (Fig. 1*c*). We found that the marmoset connectivity weights are highly heterogeneous, spanning 5 orders of magnitude, and are log-normally distributed (Fig. 1*d*), similar to macaque monkey (Markov et al. 2011, 2014a; Ercsey-Ravasz et al. 2013).

### The Connectome is Dense, Heterogeneous, and has High Specificity

In the graph theory framework, the cortex can be considered as a network where nodes correspond to areas, and edges to the connections between them. To characterize the network properties of the marmoset cortex, we considered the edge-complete $(N\times N=55)$ subnetwork, for which all inputs and outputs are known. This corresponds to approximately half of the full mesoscale connectome of this species (55/116 = 47.4%).

The interareal network density $\rho =M/N(N-1)$, defined as the fraction of existing connections $(M)$ to all possible ones, was found to be 62.43%. Even though the network is dense (Fig. 2*a*), there is high heterogeneity in the number of inputs and outputs of an area, as shown by its broad in- and out- degree normal distributions (Fig. 2*b*). Early analyses of cortico-cortical connectivity emphasized the reciprocity of connections as a prominent property (Felleman and Van Essen 1991). In the edge complete network of the marmoset connectome 50.3% are reciprocal connections, 24.24% are unidirectional, and 25.45% are absent in both directions (similarly in macaque with densities 52.71%, 26.26%, and 24.26% respectively). Although reciprocal connections are the most abundant, as well as stronger on average than the unidirectional (Supplementary Fig. 7a), bidirectionally absent connections are overrepresented when compared with those in an average random network that has same in- and out- degrees (and hence density), whereas unidirectional connections are underrepresented (Fig. 2*c*, left). Similar conclusions are reached when 3-node motifs, which are basic network building blocks (Milo et al. 2002), are considered (Supplementary Fig. 7*b*). The motifs that are overrepresented in the marmoset connectome are those that include reciprocally present and absent connections (Fig. 2*c*, right). In addition, these appear more often (data/random ratio > 2) than 2-node motifs (data/random ratio < 2). Finally, using a measure of functional similarity between any pair of areas defined by the degree of their shared inputs or outputs (Song et al. 2014), the more functionally related 2 areas are, the more likely they are to be connected (Fig. 2*d*).

Another network feature used to characterize the structure of heterogeneous dense networks is cliques, which are subnetworks of fully interconnected areas (Ercsey-Ravasz et al. 2013; Horvát et al. 2016; Goulas et al. 2019). The proportion of cliques of any size in the marmoset connectome is much higher than that of a random network with same in- and out- degree sequences, indicating high specificity (Fig. 2*e*, left). The largest clique size is 17, and there are 5 such cliques formed by overall 20 areas (Fig. 2*e*, right); these define the so-called core of the connectome, which has 98.42% density. This is broadly compatible with the core reported in Goulas et al. 2019, with small differences being due to the use of the updated dataset (Majka et al. 2020) in the present analysis. The remaining areas constitute the so-called periphery, which form a subnetwork with density 44.29%. The density of the connections between core and periphery areas is 69.07%. The weights of the connections between areas within the core, and within the periphery, are found to be stronger than those between core- and periphery (Supplementary Fig. 1*a*). Areas of the putative default mode network (DMN), including those in the posterior parietal cortex (PGM, PG, OPt, AIP), posterior cingulate cortex (A23a, A23b), and dorsolateral prefrontal cortex (A8aD, A6DR) lie in the core structure, but not those in the medial prefrontal cortex (A24d, A32, A32V), reflecting recent studies in the marmoset (Buckner and Margulies 2019; Liu et al. 2019).

### F‌F **Projections Tend to be Stronger than** FB **Projections**

The structural connectivity of the mammalian cortex is characterized both by the weights of connections between areas and by their laminar organization. A structural hierarchy of the macaque cortex has been defined based on the laminar spatial profile of the connections, according to which ascending (FF) pathways originate primarily from the supragranular layers and target layer 4 of the target area; conversely, descending (FB) pathways originate mostly from the infragranular layers, and target supragranular and infragranular layers (Rockland and Pandya 1979). This led to a hierarchical organization of the cortex (Felleman and Van Essen 1991; Barone et al. 2000; Markov et al. 2014b; Chaudhuri et al. 2015). A similar description of information flow has been proposed based on the architectonic type of each area leading to a structural model of the cortex that connects connectivity to evolution, and development (Barbas 2015; Garcia-Cabezas et al. 2019).

In this framework, the global hierarchical organization can be computed based on the percentage of supragranular neurons involved in the different connections: FF connections are formed by high percentages of supragranular neurons in source areas, and FB connections by low percentages. We calculated the percentage of supragranular labeled neurons (SLN) for a given tracer injection as the number of labeled neurons above layer 4 divided by the total number of all labeled neurons found in the source area (Fig. 3*a*). When multiple injections were placed in the same area, the SLN was calculated as the weighted average value for the injections that revealed a given connection (Fig. 3*b*). We found that marmoset cortical connections span the entire range (0–1) of possible SLN values (Fig. 3*c*), similar to the macaque SLN distribution, which was shown to be approximately uniform (Markov et al. 2014b). Furthermore, we found that predominantly FF $(\mathrm{SLN}>0.5)$ connections tended to be stronger than predominantly FB projections (higher mean FLN by a factor of 2; Fig. 3*d*). The trend is similar in the macaque, as was shown in Markov et al. 2014b, but less pronounced (Supplementary Fig. 8).

### Laminar Organization of Connections Reveals Modal Hierarchies

To characterize the global hierarchy, we followed a framework used in previous studies (Markov et al. 2014a; Chaudhuri et al. 2015) in which hierarchical indices are assigned to each area such that for any pair of cortical areas the difference of their hierarchical indices predicts the SLN of their projection (Materials and Methods). Ordering these indices in Figure 4*a* reveals the hierarchy of the edge-complete network. Sensory areas are situated at the bottom of the hierarchy providing FF inputs to most other areas, and association areas form mostly FB projections (Fig. 4*a*). Furthermore, motor areas tend to be concentrated above posterior parietal and prefrontal areas (similarly in macaque cortex but to a lesser extent, Supplementary Fig. 2*a*), with the ventral premotor cortex being situated at the top of the hierarchy.

This hierarchical ordering aligns the cortical areas based on SLN, but does not capture topological properties of the connectivity, for example, the fact that many connections do not exist. This caveat is mitigated by considering also the core-periphery structure. In this representation, functionally related sensory areas are grouped together in the wings of a “bowtie” graph, with the core occupying the center (Markov et al. 2013) (Supplementary Fig. 9). The core structure receives effectively stronger FF inputs from visual areas, somatosensory and medial prefrontal areas and effectively stronger FB projections from auditory, posterior parietal, and motor areas.

The bowtie graph is based on topological binary connections. By including the weights of connections, we can extract information about the overall underlying cortical architecture, represented by a polar plot on a 2-dimensional plane (Chaudhuri et al. 2015). In this representation (Fig. 4*c*), both the weight of the connections (inverse of the angle between areas) and their hierarchical index (distance from the edge) are considered. Areas deemed to correspond to low hierarchical levels appear in the periphery of the graph, with hierarchical level progressing toward the center. This analysis shows that the marmoset cortical network is better described by a series of modal hierarchies, which converge toward a region formed by multimodal and high-order premotor areas. For example, a hierarchy of visual areas is revealed, grouped together in one quadrant of the plot, which progresses toward parietal areas and frontal areas that are involved in visual cognition (e.g., LIP, Opt, PGM, the frontal eye field [area A8aV] and ventrolateral prefrontal area A47L). The somatosensory and motor areas form another hierarchical grouping in a different quadrant, and areas that are involved in visuomotor integration and planning lie between the visual and somatosensory/motor clusters (e.g., PEC, MIP, and AIP). Finally, the auditory cortex forms a third grouping in a separate quadrant of the plot, with multisensory areas of the temporal lobe (e.g., caudal TPO, PGa-IPa) separating them from visual areas. Interestingly, the prefrontal areas that align best with the auditory hierarchy are those in which single-unit activity is related to orientation to sounds in space (e.g., area A8aD) and processing of vocalization sounds (e.g., areas A32/A32V). Areas associated with the DMN (Buckner and Margulies 2019; Liu et al. 2019) tend to be located near the center of the diagram (e.g., A23a, A6DR).

### Microstructural Properties of the Cortex Reflect the Hierarchical Organization

An emerging theme of large-scale cortical organization is that biological properties of cortical areas show spatial gradients that correlate with hierarchical level (Chaudhuri et al. 2015; Burt et al. 2018; Fulcher et al. 2019; Wang 2020). Microscale properties are correlated with macroscale connectivity patterns (Scholtens et al. 2014). In addition, previous studies in the macaque (Elston and Rosa 1998) suggested that hierarchical processing is associated with progressively greater numbers of synaptic inputs (leading to greater allocation of space to neuropil, hence lower neuronal densities). The analysis illustrated in Figure 5 lends support to this hypothesis for the marmoset cortex, in an analysis that combines the newly quantified hierarchical rank and estimate of spine counts on the basal dendritic trees of layer III pyramidal neurons (see Supplementary Table 4 and Supplementary Figure 6 for sources of data and harmonization of nomenclatures). The average spine counts are highly correlated with hierarchical level (*r* = 0.81). This correlation is as high as the average spine count correlation with the spatial location of the areas along the rostrocaudal axis (Supplementary Fig. 10*a*,*b*), which was suggested as another strong predictor of network architecture based on developmental considerations (Cahalane et al. 2012). This suggests the rostrocaudal axis as proxy for hierarchy.

Given that spine count increases along the hierarchy, while neural density decreases (Supplementary Fig. 10*c*, left), it could be that the spine density (spine count/number of neurons in a unit of cortex volume) is invariant across cortical areas. However, our data show that the spine count increases as the inverse cube of neural density (Supplementary Fig. 10*d*), thus supporting previous suggestions that hierarchical processing is associated with progressively greater numbers of synaptic input. Note of course that the spine count corresponds to the spines of the basal dendrites of the average layer III pyramidal neuron, whereas the neural density to all neurons. It has been suggested that the number of neurons in a cortical column is a better correlate of hierarchical processing (Atapour et al. 2019), but we found weaker correlations (Supplementary Fig. 10*c*, right). Finally, our results support the proposal that both neural density and spine count are good predictors of the laminar origin of projections (Beul and Hilgetag 2019), since they are highly correlated, and both correlate strongly with the hierarchy.

### Spatial Embedding of the Connectivity

Parcellated areas in a neocortical network are traditionally considered as nodes of a topological graph, without considering their spatial relationships. However, in addition to its statistical and topological properties, the cortical connectome is spatially embedded. It has been proposed that the metabolic cost of sending information from one area to another increases with distance, being reflected in an EDR (Ercsey-Ravasz et al. 2013) according to which the projection lengths decay exponentially. Incorporation of this attribute in generative models of the connectome helps explain network properties such as efficiency of information transfer, wiring length minimization, 3-motif distribution, and the existence of a core (Ercsey-Ravasz et al. 2013; Song et al. 2014; Horvát et al. 2016; Wang and Kennedy 2016).

**Figure 6:**
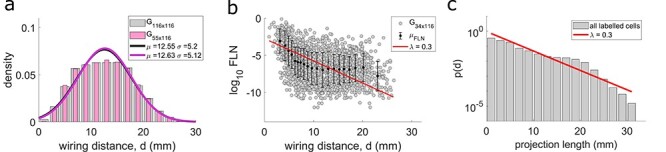
Exponential distance rule. (*a*) Distribution of the interareal wiring distances among all 116 areas (gray bars) and between the target-source pairs of areas (pink bars). Bin size is 2 mm. Solid lines are Gaussian fits to the data. The normal distributions for these 2 samples of data indistinguishable (2-sided 2-sample Kolmogorov–Smirnov test: $p=0.97$). (*b*) The base 10 logarithm of the fraction of the extrinsic labeled neurons $({\log}_{10}\mathrm{FLN})$ as a function of interareal wiring distance. Black dots and error bars are the mean and standard deviation within a window of 173 data points (bin size is 20) and the red plot is the same as in (*c*). (*c*) The histogram of the projection lengths of all labeled neurons (both intrinsic (551 664 labeled neurons) and extrinsic (1 414 364 labeled neurons), in total 1 966 028 labeled neurons). Bin size is 2 mm and the bars are the counts of the projection lengths lying in the bin size divided by the total number of the projections. The red line is a linear fit to the base 10 logarithm values of the histogram $({\log}_{10}(p(d))=-0.1295\ {\log}_{10}(\mathrm{projection}\ \mathrm{length})-0.0262$ giving $p(d)=c{e}^{-\lambda d},\lambda \approx 0.3,c\approx 0.94$, where *d* is the projection length).

To study the spatial organization of the marmoset cortical connectome we used estimates of the distances between the barycenters of cortical areas, obtained with an algorithm that simulates white matter tracts connecting points on the cortical surface (Majka et al. 2020). In agreement with observations in other species [macaque (Ercsey-Ravasz et al. 2013), mouse (Horvát et al. 2016)] we found that the wiring distances are normally distributed (Fig. 6*a*). The distribution of the wiring distances of the pairs for which we have connectivity data ($55\times 116$ matrix, Fig. 1*c*) overlaps with that for all the cortical areas ($116\times 116$), indicating that this subset of data is representative of the full network.

We show that the more distant two areas are, the lower the probability of a projection from one to the other, as evidenced by reduced FLN (Fig. 6*b*). However, analyses based on FLN are anchored on estimates of the borders of cytoarchitectural areas, which in most cases are imprecise (Rosa and Tweedale 2005). This limitation can be overcome by measuring the wiring distance between each labeled neuron and the corresponding injection site, in an area-independent manner. Based on the stereotaxic coordinates of each injection site and labeled neuron, we calculated the shortest distance across the white matter corresponding to each connection detected in the database (Majka et al. 2020). This included the projection lengths of 1 966 028 labeled neurons, including both those estimated to be in the same area that received the injection (intrinsic connections) and those in other areas. The cost of each neuron to project to longer distances can then be expressed by the distribution of the projection lengths of all the retrogradely labeled neurons (Fig. 6*c*), which illustrates the probability of a projection length *d*, irrespectively of the areas involved.

As in previous studies in macaque and mouse (Ercsey-Ravasz et al. 2013; Horvát et al. 2016; Wang and Kennedy 2016), we found that the histogram of axonal projection lengths approximately follows an exponential decay (Fig. 6*c*) with decay rate $\lambda =0.3$; that is, the probability of a projection of length *d* is given by $p(d)=c{e}^{-\lambda d}$. Approximating the projection probability with the FLN (*p*(*d*)$\approx$FLN), it was analytically shown that we can replicate the log-normal distribution of the connectivity weights (Ercsey-Ravasz et al. 2013). In the marmoset, this approximation is also valid since the decay of the probability of projection lengths (as seen by the decreasing trend of the average values; black dots and the corresponding error bars in Fig. 6*b*) agrees with the ${\log}_{10}\mathrm{FLN}$ decay with wiring distance (red plot in Fig. 6*b*). We note that the scatter in Figure 6*b* is large, but it is comparable to the scatter in macaque and mouse where the EDR trend has been reported. In addition, the curvature of the average ${\log}_{10}\mathrm{FLN}$ and the small deviation of projection lengths from the log decay at distances around 20 mm may suggest the possibility of a more complex relation of the projection lengths distribution. Nevertheless, we showed that the projection lengths distribution can be approximated by the EDR, which is an overall statistical property of the cortex.

### Exponential Decrease in Wiring Distance Scales with Brain Volume

**Figure 7:**
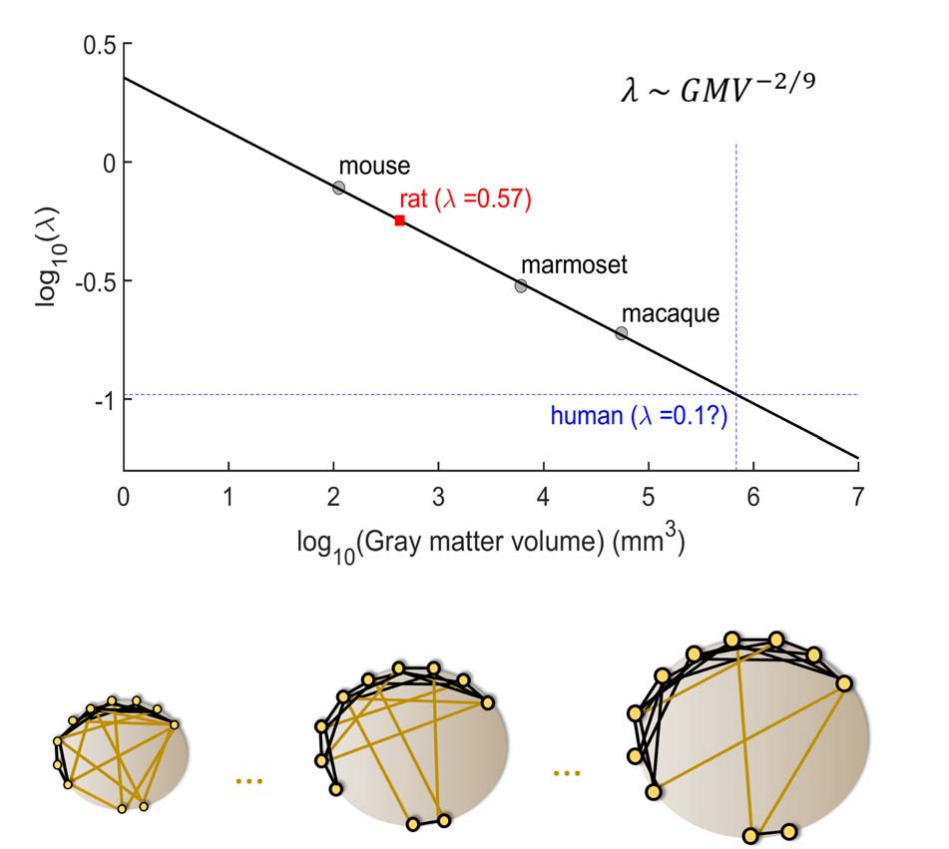
Cortical-connectivity spatial length as a function of brain size: extrapolation to humans. Top: The base 10 logarithm of the decay rate of the EDR (*λ*) of the mouse, marmoset, and macaque, computed in the same way in all 3 cases. The plot is a linear fit on these 3 points with a slope of $\approx -2/9$  $({\log}_{10}(\lambda )=-0.2290\ {\log}_{10}(\mathrm{gray}\ \mathrm{matter}\ \mathrm{volume})+0.3559)$. The red square is the predicted value of the decay rate of the EDR of the rat which is validated by indirect methods (Noori et al. 2017) of computing it, and the intersection of the blue dotted lines is the extrapolation of the decay rate of spatial dependence of cortico-cortical connectivity in the human species. Bottom: A schematic representation of the decrease of long-range connections as the gray matter gets bigger, showing that the bigger the brain the more local the connectivity.

Finally, we address the question of how the EDR of cortical connectomes scales across species. It was previously shown that the decay rate of the EDR is larger in macaque than in the mouse following normalization of the distances by the average interareal wiring distance (common template) (Horvát et al. 2016). This suggests that the larger the brain, the fewer are the long-range connections linking different cortical systems (as shown schematically in Fig. 7, bottom).

This principle was upheld in our analysis of the marmoset cortex (Supplementary Fig. 5), where the decay rate in the marmoset shows an intermediate value. Plotting the present data relative to the previous studies in which similar methods were used (macaque and mouse) as a function of the gray matter volume, we found that the decay rate of the EDR $(\lambda )$ scales with gray matter volume following a power law (Fig. 7, top) with an exponent of $-2/9$. Given that $\mathrm{WM}\sim{\mathrm{GM}}^{4/3}$ (where $\mathrm{WM}$: white matter volume and $\mathrm{GM}:$ gray matter volume) (Zhang and Sejnowski 2000), and if we define the linear dimension $d$ as ${\mathrm{WM}}^{1/3}$, the decay rate of the axonal projections scales with the inverse square root of the white matter linear dimension, $\lambda \sim{d}^{-1/2}$. It is surprising that the dependence of the characteristic spatial length for EDR is slower than the linear dimension of the white matter. The implication is that the interareal connections become more spatially restricted in a larger cortex, which presumably is desired for increasing complexity of modular organization. This power law predicts the decay rate for the rat cortex to be ${\lambda}_{\mathrm{rat},\mathrm{predicted}}\sim 0.57\ {\mathrm{mm}}^{-1}$, which is validated by that estimated indirectly for the rat (${\lambda}_{\mathrm{rat},\mathrm{data}}=0.6\ {\mathrm{mm}}^{-1}$) by fitting the EDR to properties of the rat connectome (Noori et al. 2017). Finally, using this relation we can extrapolate the decay rate of the projection lengths of the human cortical connectome, which is predicted to be ${\lambda}_{\mathrm{human},\mathrm{extrapolated}}\sim 0.1\ {\mathrm{mm}}^{-1}$ (Fig. 7). We should note that (Mota et al. 2019) have challenged the universal scaling of the white matter volume with the gray matter volume across mammalian species (Zhang and Sejnowski 2000). However, the differences between clades they reported are small, suggesting that they would not significantly impact on this result.

## Discussion

We studied the statistical, architectonic, and spatial characteristics of the marmoset cortical mesoscale connectome, based on the largest available dataset for cellular-level connectivity in a primate brain (http://marmosetbrain.org). Our main findings are 3-fold. First, the marmoset cortical connectome is highly dense at the interareal level, characterized by high heterogeneity of inputs and outputs as well as connectivity weights. Moreover, connections are highly specific, evaluated by the presence and absence of reciprocal connections, the dependence of connections on the functional similarity, the distribution of cliques, and the core-periphery structure. Second, based on the laminar origin of the projections, we also provided here, for the first time, a quantified hierarchical structure of the marmoset cortex which, in conjunction with connectivity weights, revealed parallel processing streams of sensory and association areas, which converge toward a highly connected core. Third, interareal connections obey the same approximate EDR for marmoset as for macaque and mouse cortex. Intriguingly, the characteristic spatial length of the interareal connections revealed an allometric scaling rule as a function of the brain size among mammals, leading to a predicted value for the human cortex and that of other species, which can be tested experimentally.

### Scaling across Species

Current estimates of anatomical connectivity in the human brain are based on diffusion tensor magnetic resonance imaging tractography, which has been shown to be only modestly informative in studies involving direct comparisons with gold-standard neuronal tracer data (Donahue et al. 2016). Hence, comparative studies that provide insight on the scaling rules of connectivity in nonhuman primates are essential to provide valuable insight on how the increase in brain volume in human evolution is likely to have affected network properties.

Marmosets are New World monkeys, a group that shared a last common ancestor with macaques and humans approximately 43 million years ago (Perelman et al. 2011). In contrast, the divergence between rodents and primates is estimated to have occurred around 80 million years ago (Foley et al. 2016). Here, we provide evidence toward the common and species-unique cortical connectivity properties. We show that the connectivity weights of the marmoset are log-normally distributed, similar to those in the macaque (Markov et al. 2011, 2014a; Ercsey-Ravasz et al. 2013) and mouse (Wang et al. 2012; Oh et al. 2014; Gămănuţ et al. 2018), indicating that this is a general property of the mammalian cortico-cortical connections. In addition, the range of connectivity weights encompasses 5 orders of magnitude, with a gradual increase in mean connectivity weight as the brain gets smaller (Supplementary Fig. 11). Current estimates indicate ~ 40 cytoarchitectural areas in the mouse brain, excluding subdivisions of the hippocampal formation (Oh et al. 2014), 116 in the marmoset (Paxinos et al. 2012), and 152 in the macaque (Saleem and Logothetis 2012). Thus, one possibility to account for the above observations is that the dilution of connectivity reflects a gradual redistribution of connections across a larger number of nodes, in larger brains. To test this, we compared the density of the edge-complete graph for available data obtained in the macaque, mouse, and marmoset. Perhaps surprisingly, the results (Supplementary Fig. 12) revealed that this is not the case: the density of the cortico-cortical graph is very similar in macaque and marmoset, despite substantial differences in cortex mass and number of cytoarchitectural areas. The above conclusion is robust across the application of different thresholds for what is considered a valid connection, an analysis that minimizes the possibility of artifacts related to assignment of cells to adjacent areas due to uncertainty in histological assessment of borders. Both primates differ from the mouse, in which the graph density is much higher irrespective of the threshold applied (Supplementary Fig. 12). Thus, although one may expect an inverse relationship between connection densities and brain volume (Ringo 1991; Gămănuţ et al. 2018), our results also suggest a difference between primates and rodents. The present observations are in line with the suggestion that the lower graph density and lower relative fraction of long-range connections in primate brains, as opposed to rodent brains, are likely to render the former more susceptible to disconnection syndromes such as schizophrenia and Alzheimer’s disease (Horvát et al. 2016). This has likely implications for the design of translational research aimed at alleviating human mental health conditions (see also Van Essen and Glasser 2018; Buckner and Margulies 2019).

Since FLN can be viewed as area-dependent probability of interareal connection, our data suggest scaling of the weights of cortico-cortical connections in such a way that the larger the brain, the larger the proportion of very sparse connections. The extrapolation of these results suggests that the human cortex is likely to be characterized by a comparatively more distributed architecture, showing an even larger proportion of numerically sparse connections. Further, given the scaling of the EDR with brain size, the data also predict that the larger human brain likely shows a more marked predominance of local connectivity, resulting in a larger number of subnetworks linked by a core (Fig. 7, bottom), as previously suggested (Buckner and Krienen 2013; Horvát et al. 2016).

### Comparative Aspects of the Cortical Network Properties

We have shown that both the marmoset and macaque connectomes exhibit high and similar density. In addition, their in- and out-degree distributions, and the 2- and, 3-node motif distributions, are similar (Supplementary Figs 7*c*,*d* and 13), indicating conserved topological properties independent of the brain size. Another conserved property is that functionally related areas are most likely to be connected. This is related to the wiring distance, with the decay rate of the probability of connections of the marmoset following closely that of the macaque (Supplementary Fig. 4) (Song et al. 2014). Furthermore, the marmoset cortex has more fully interconnected large subnetworks (clique size > 6) in comparison with the macaque (Supplementary Fig. 1*b*), supporting the view that the smaller the brain, the higher the interconnectivity, even among primates. Finally, both the marmoset (Fig. 2*e*, right) and the macaque (Ercsey-Ravasz et al. 2013) show a similar core structure consisting mainly of association areas.

### Variability of Injections

Repeated injections in what are currently considered single cytoarchitectural areas (Supplementary Table 3) revealed variability in their patterns of afferent connections (Supplementary Fig. 14*a*). In similar studies of the macaque and mouse connectomes (Markov et al. 2011, 2014a; Gămănuţ et al. 2018), it has also been shown that the connectivity weights are variable, but less so in the mouse compared with the macaque. One interpretation of these observations is that connectivity patterns across individuals are more consistent in smaller brains, which have fewer subdivisions. This could result from differences in postnatal refinement of patterns of connections in different individuals, which could be more significant in light of more complex behaviors and interactions with the environment. However, other potential sources are within area variability (e.g., differences between the connections of regions serving foveal and peripheral vision (Palmer and Rosa 2006b), and between parts of the motor cortex related to limb and face movements (Burman et al. 2014), hemispheric differences (a subject for which little is known in nonhuman primates), and those related to the characteristics of individual injections (Majka et al. 2020). In previous studies, there was deliberate targeting of the same part of the area across subjects, whereas in our sample the injections covered different parts across and within subjects (Supplementary Table 2). Thus, the macaque samples were inherently homogeneous, whereas ours may better reflect the real variability of connections of cytoarchitectural areas. In order to assess this variability thoroughly, a larger statistical sample is required. Nevertheless, the qualitative results based on the weighted values (the FLN and SLN) should be robust to appropriately applied thresholds based on variability across injections. As an example, we show that the decay of the FLN with distance is not affected by considering only the FLN with smaller coefficient of variation across injections in the same target area (Supplementary Fig. 14*b*).

Another potential source of variability is the inclusion of injections that crossed the estimated borders between 2 cytoarchitectural areas (Majka et al. 2020). This is intrinsically difficult to evaluate in many cases, since the borders of many areas are not sharp (Rosa and Tweedale 2005). Here, injections were assigned to areas based on estimates of how close the injection was to the border and of percentages of the injections contained in each area (see Discussion and Supplementary Table 1 in Majka et al. 2020). However, considering only the injections estimated to be confined at least 80% within the target area (120 injections in 50 target areas; Supplementary Fig. 15*b*), or injections confined 100% within a target area (79 injections in 34 target areas; Supplementary Fig. 15*c*) does not substantially affect our conclusions (Supplementary Figs 12, 16, and17).

### Hierarchical Organization

Our analysis of the hierarchical structure of the marmoset cortex indicates that some of the premotor areas, including the ventral premotor cortex (area 6Va), are situated at the top of the hierarchy (Fig. 4). In other words, such areas form a large proportion of projections with characteristics of FB projections (low SLN). This appears in conflict with the data so far obtained in the macaque, in which association areas such as the prefrontal cortex lie at the highest hierarchical levels (Chaudhuri et al. 2015) (Supplementary Fig. 2). In addition, the intermediate functional groups were less clearly differentiated. To some extent, this may simply reflect differences in the availability of data. For example, to date, data from the macaque cortical network do not include injections in ventral premotor cortex (areas F4/F5). Conversely, data obtained in the rostral part of the superior temporal polysensory cortex (area TPO, or “STPr” in the macaque) are not available for the marmoset, where only the caudal part of TPO was injected. Functionally, if the final goal for the cortex is to generate behaviors, it could be expected that the flow of information culminates in motor areas involved in higher-order planning of sequences of movements, such as A6Va and A6DR, which integrate stimulus-initiated and internally initiated information, toward generation and evaluation of actions. The ventrolateral posterior region of the frontal lobe has expanded considerably in human evolution (Chaplin et al. 2013), including the emergence of Broca’s area in the human brain, suggesting a high-order station for integration of information from various sources, toward the generation of complex behavior. However, including the orthogonal dimensions of the presence/absence of connections and weights of connections enabled us to obtain a more comprehensive insight into the interareal architecture, where there are parallel sensory streams associated with different higher-level areas. Given the larger number of target areas and pathways studied, this configuration appears clearer than in previous studies of the macaque cortex (Chaudhuri et al. 2015).

### Future Directions: Large Scale Models of the Mammalian Brain

One of the principal open problems in systems neuroscience is understanding the structure-to-function relationship. Toward achieving this goal, there is an increasing interest in modeling whole-brain dynamics, as opposed to modeling individual areas. Early models incorporated neuroimaging-based structural connectivity data (Cabral et al. 2017; Demirtaş et al. 2019). The major advantage of these studies is that they can model the human brain based on noninvasive data. However, there are important caveats to these modeling studies including the low resolution of the imaging techniques and, critically, directionality of the resulting structural connectivity matrix. On the other hand, we show that the reciprocity of connections, and absence of connections, are prominent attributes of the connectome (Fig. 2*c* and Supplementary Fig. 7), which begs the question whether and how unidirectional connections are important to functions. More recently, there have been a series of large-scale network models that incorporate the weighted and directed structural connectivity obtained via retrograde tracing methods, as well as the hierarchical organization of the areas based on the laminar distribution of the projections (Chaudhuri et al. 2015; Mejias et al. 2016; Joglekar et al. 2018). The results presented here provide a foundation for a future large-scale network model of the marmoset cortex, which will serve to clarify the computations underlying marmoset brain function and behavior. Ultimately, the increased knowledge of the scaling properties of the cortical cellular network in nonhuman primates, together with large scale in silico models of other mammals and comparisons of data obtained with neuroimaging techniques (Hori et al. 2020), will help bridge the gap between animal models and humans, leading to a better understanding of normal and pathological functions, as well as brain evolution.

## Supplementary Material

supplementary_rereview_May2021_bhab191

## Notes

The authors would like to thank Henry Kennedy, Kenneth Knoblauch, and Maria Ercsey-Ravasz for discussions and Jorge Jaramillo for feedback on the manuscript. *Conflict of Interest:* None declared.
